# Hypoglycemic Effects of Crude Polysaccharide from *Purslane*

**DOI:** 10.3390/ijms10030880

**Published:** 2009-03-02

**Authors:** Fayong Gong, Fenglin Li, Lili Zhang, Jing Li, Zhong Zhang, Guangyao Wang

**Affiliations:** 1 Department of Food Science, XiChang College, Sichuan Province, 615000, P. R. China; 2 Department of Bioengineering, Jilin Agricultural Science and Technology College, Jilin Province, 132101, P.R. China; E-Mail: swgclifenglin@sina.com (F.L.); zhanglili1975@sina.com (L.Z.)

**Keywords:** Diabetes, *Purslane*, polysaccharide, hypoglycemia

## Abstract

The effects of crude polysaccharide from *Purslane* (CPP) on body weight (bw), blood glucose, total cholesterol (TC), high-density lipoprotein cholesterol (HDL-c), triglyceride (TG) and serum insulin levels were studied in diabetes mellitus mice. CPP treatment (200, 400 mg/kg bw) for 28 days resulted in a significant decrease in the concentrations of fasting blood glucose (FBG), TC and TG. Furthermore, CPP significantly increased the concentration of HDL-c, body weight and serum insulin level in the mice. In addition, according to acute toxicity studies and single cell gel electrophoresis analysis, CPP did not produce any physical or behavioral signs of toxicity. More significantly, our data demonstrated CPP exhibited the best effects at the dose of 400 mg/kg bw. The above results suggest that CPP can control blood glucose and modulate the metabolism of glucose and blood lipids in diabetes mellitus mice, so we conclude that CPP should be evaluated as a candidate for future studies on diabetes mellitus.

## 1. Introduction

*Purslane* (Little Hogweed; Chinese name: Ma-Chi-Xian) is a grassy plant with small yellow flowers and stems sometimes flushed red or purple, which grows widely in different areas of the world including the north of China [1,2]. The plant contains many biologically active compounds, including free oxalic acids, alkaloids, omega-3 fatty acids, coumarins, flavonoids, cardiac glycosides, and anthraquinone glycosides [3,4]. *Purslane* is considered a type of common weed, but it can be eaten as a potherb without any side effects. Moreover, *Purslane* is known in folk medicine in some parts of China as a hypotensive and antidiabetic [5,6,7]. Though there is no scientific evidence to support the antidiabetic effects of *Purslane*, people continue to use it in the treatment of this condition. The objective of this study was to extract crude polysaccharide(s) from *Purslane* and assess the hypoglycemic effects of these constituents with animal tests for the use of this plant in the treatment of diabetes.

## 2. Results and Discussion

### 2.1. Acute toxicity studies

Acute toxicity studies revealed no obvious symptom of toxicity of CPP or any significant changes in general behavior in mice. There was no lethality or any toxic reactions found at any of the doses selected through the end of the study period.

### 2.2. Effect of CPP on body weight in mice

Alloxan-induced diabetic mice exhibited loss of body weight [8,9]. Before embarking on the experiments, all the groups had no significant difference in body weight (*P >0.05*) (Table 1).

A significant (*P < 0.05*) decrease in body weight was detected in the DC, DLCPP and DHCPP groups as compared to the normal control group from 7 days after alloxan injection. However, the body weights in the DHCPP group were significantly (*P < 0.05*) and dose-dependently increased as compared to those of the diabetic control from 14 days after administration. In the DGLI group, a significant (*P < 0.05*) increase in body weight as compared to the DC group was also detected from 7 days after administration. The results are shown in Table 1. The present study showed that the body weight was significantly decreased in the diabetic control group as compared to the normal control group within 28 days. However, the treatment with CPP for 28 days improved the body weight loss.

### 2.3. Effect of CPP on fasting blood glucose levels in mice

Effective control of the blood glucose level is a key step in preventing or reversing diabetic complications and improving the quality of life in both Type 1 and Type 2 diabetic patients [10,11]. The alloxan-induced diabetic mice exhibited hyperglycemia. In the diabetic groups, a significant (*P*< 0.05) increase in FBG was detected, as compared to the normal control group, but these abnormal increases in blood glucose levels significantly (*P* < 0.05) and dose-dependently decreased in the CPP-administered groups as compared to the diabetic control group from 7 days after administration. On the 28th day, blood glucose levels in the DLCPP and DHCPP groups had decreased by 36.0% and 62.9%, respectively. In the DGLI group, the decrease was also significant (*P* < 0.05) from 7 days after administration. The NC and DC groups did not show any significant variation on the blood glucose level throughout the experimental period (p > 0.05). The results are shown in Table 2.

The present study showed that alloxan-induced diabetic mice presented obvious hyperglycemic symptoms, but CPP produces a significant antihyperglycemic effect when administered orally to alloxan-diabetic mice. The dosage of 400mg/kg is more effective than that of 200 mg/kg.

### 2.4. Effect of CPP on blood lipids levels in mice

Diabetes mellitus is usually complicated with hyperlipoproteinemia. The present results showed that the TC and TG levels were significantly elevated in the diabetic control group as compared to the normal control group (*P* < 0.05), and serum HDL-c level, a friendly lipoprotein, was decreased in diabetic control group as compared to the normal control group (*P* < 0.05). After supplementation with CPP and glibenclamide, the alteration in lipid metabolism was partially attenuated as evidenced by decreased serum TG and TC levels and by increased HDL-c concentration in diabetic mice. The response was better in the DHCPP group compared to the DLCPP group which is comparable to that of the DGLI group. The results are shown in Table 3.

The serum TC and TG were decreased significantly in diabetic mice after CPP supplementation. These effects may be due to low activity of cholesterol biosynthesis enzymes or low level of lipolysis which are under the control of insulin [12].

### 2.5. Effect of CPP on blood serum insulin levels in mice

**Figure 1.**
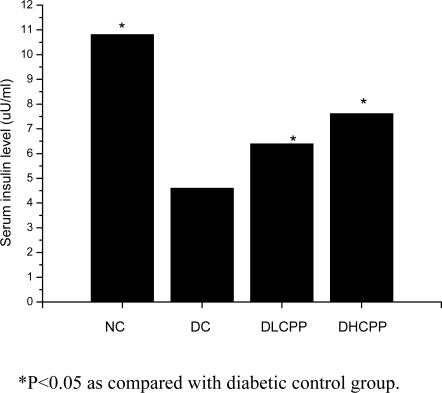
Effect of CPP on serum insulin level in mice. *P<0.05 as compared with diabetic control group.

Serum insulin levels of the normal control group were higher than those of the diabetic control group, which indicated that alloxan damaged the pancreas islet cells. After 28 days of the CPP supplementation to the mice, there was a significant elevation in serum insulin level as compared to the diabetic control group (*p < 0.05*), which implied that treatment with CPP improved the insulin secretion on diabetic mice. In the DHCPP group, the insulin level was higher than that of the DLCPP group. The results implied that CPP improved the function of islet cells and stimulated the insulin secretion. The results are shown in Figure 1.

Alloxan could damage pancreatic β cells, resulting in a decrease in endogenous insulin secretion, which decreased utilization of glucose by the tissues consequently. In this study, we have observed that CPP increased the concentration of serum insulin in alloxan-induced diabetic mice. The possible mechanism of action of CPP could be correlated with promoting insulin secretion by closure of K^+^-ATP channels, membrane depolarization and stimulation of Ca^2+^ influx, an initial key step in insulin secretion[13]. Additional studies are needed to address this hypothesis.

## 3. Experimental Section

### 3.1. Plant materials

*Purslane* was collected in Sichuan Province in July and the material was identified by Mr. Wang Guang-Yao, a botanist from the Jilin Agriculture Science and Technology College. A voucher specimen has been deposited in the herbarium of the Jilin Agriculture Science and Technology College. Fresh and intact *Purslane* dried in the shade was chosen as experimental material.

### 3.2. Drugs and reagents

Alloxan was purchased from Sigma Co. (USA). Glucose Analyzer and strips were purchased from Roche Diagnostic Co. (USA). Reagents for total cholesterol (TC), triglyceride (TG), high-density lipoprotein cholesterol (HDL-c) were obtained from Beijing Chengxinde Biochemistry Reagent Company (Beijing, P.R. China). Reagents for serum insulin was purchased from Adlitteram Diagnostic Laboratories Co. (USA).

### 3.3. Preparation of crude polysaccharide from Purslane (CPP)

The shade dried *Purslane* was crushed in an electrical grinder and then powdered, 1,000 g of this powder was immersed in tenfold dH_2_O and boiled at 100 °C for 9 h [14–16], and then the water extract was collected. The process was repeated, and the extracts were combined and concentrated on a vacuum rotary evaporator at 60 °C. The concentrated solution was precipitated by addition of four times its volume of volume 80% ethanol and the precipitate was washed in turn with 100 % ethanol, 100 % ether and acetone. Crude polysaccharide from *Purslane* (CPP) was obtained by vacuum drying [14,15]. A Unico-7200 spectrophotometer (Unico Co., Shanghai, P.R. China) was used to determine the content of polysaccharides in the above extracted product at 490 nm [15,16]. The polysaccharide content was calculated using the following linear equation based on the calibration curve:

$$\text{Y}=3.809\times 10^{-2}+1.321\text{X},\text{r}=0.9998$$

where Y is the absorbance and X is the polysaccharide content.

### 3.4. Animals and diets

Male mice of original Kun-ming strain, approximately 18 to 22 g, were obtained from the Animal Department of Beijing Institute of Traditional Medical and Pharmaceutical Sciences. The animals were housed in a room maintained at 22 °C to 25 °C with relative air humidity of 50 % to 70 % controlled room under a 12 h light-dark cycle, and basal diet and water were supplied *ad libitum*. Approval of this experiment was obtained from the Institutional Animal Ethics Committee of XiChang College.

### 3.5. Preparation of diabetic mice

Male mice were adapted to diet for 1 week before the experiment began [16]. After a 14 h fasting, mice were induced with a single injection of 4 % alloxan prepared freshly at a dose of 200 mg/kg bw [11,12]. Diabetes was confirmed by the determination of tail vein blood glucose levels on the third day after administration of alloxan. Mice having blood glucose levels greater than 11.1 mmol/L were considered diabetic and used for the study.

### 3.6. Experimental design

*Portulaca oleracea* L. polysaccharide (CPP) and glibenclamide (Glib) were dissolved in distilled water and were fed by gavage to mice once a day. Forty male mice were randomly divided into five equal groups as follows [17–20]:

- Normal control group (NC): normal control mice administered water daily for 28 days;
- Diabetic control group (DC): diabetic control mice administered water daily for 28 days;
- Diabetic + CPP (200 mg/kg) group (DLCPP): diabetic mice administered CPP (200 mg/kg) daily for 28 days;
- Diabetic + CPP (400 mg/kg) group (DHCPP): diabetic mice administered CPP (400 mg/kg) daily for 28 days;
- Diabetic + glibenclamide (4 mg/kg) group (DGLI): diabetic mice administered reference drug Glib (4 mg/kg) daily for 28 days.

Animals of control group, NC and DC groups were subjected to forceful feeding of 0.5 mL distilled water/100 g bw daily for 28 days to keep all the animals at same type of treatment condition in respect to CPP supplemented groups.

On the starting day, fasting blood glucose (FBG) was monitored of all the animals in each group. On the 28th day of the experiment, the mice were sacrificed by decapitation under light ether anesthesia and blood was collected from dorsal aorta and serum was separated by centrifugation for 5min and was kept at −20 °C for the biochemical assay of total cholesterol (TC), high-density lipoprotein cholesterol (HDLc), triglyceride (TG) and for serum insulin assay. TC, TG, and HDL-c were determined by enzyme methods; Serum insulin level was estimated by insulin-ELISA kit according to the manufacturer’s instruction.

### 3.7. Testing of fasting blood glucose (FBG)

During the CPP supplement for 28 days, fasting blood glucose level was measured for once every week. Blood was collected from tip of the tail vein and fasting blood glucose level was measured by using a glucose analyzer. At the same time, the body weight of each mouse was measured by balance.

### 3.8. Acute toxicity studies

Healthy mice (18–22 g) of either sex, starved overnight were divided into three groups (n=6) and were orally fed with CPP in increasing dose levels of 250, 500, 750 and 1,000 mg/kg bw The animals were observed continuously for 2 h under the following profiles [21]:

- Behavioral profile. Alertness, restlessness, irritability, and fearfulness.
- Neurological profile. Spontaneous activity, reactivity, touch response, pain response and gait.
- Autonomic profile. Defecation and urination.

After a period of 24 and 72 h they were observed for any lethality or death.

### 3.9. Statistical analysis

All results were expressed as mean ± SD and were analyzed by SPSS for Windows, version 13.0 (SPSS Inc, Chicago). The Duncan test and one way analysis of variance were used for comparisons[22,23]. The values were considered significant when P < 0.05.

## 4. Conclusions

Our research has indicated that CPP possesses antidiabetic activities and the dose of 400 mg/kg bw represents the optimal level for effecting a positive diabetic response in mice. Therefore, CPP should be considered as a candidate for future studies on diabetes. Although, in this test, the main pharmacological ingredient of CPP is polysaccharide, the further studies are in progress to clarify antidiabetic activity of the other compounds and to elucidate the molecular and cellular mechanism of CPP.

## Figures and Tables

**Table 1.** Effect of CPP on body weight (g) in mice.

Group	Number of animals	Days after dosing				
		0d	7d	14d	21d	28d
NC	8	18.99±0.60	23.45±0.57	25.39±0.57	27.56±0.57	30.22±0.85
DC	8	19.14±0.75	20.65±0.74*	22.40±1.06*	23.40±0.99*	24.41±1.51*
**DLCPP**	**8**	**19.15±1.14**	20.97±1.44*	22.93±1.53*	25.47±1.07*#	26.90±0.96*#
DHCPP	8	19.49±0.74	21.50±1.29*	23.51±0.95*#	26.01±0.65*#	27.62±1.13*#
DGLI	8	19.08±1.07	22.62±1.52#	24.97±0.98#	26.91±0.99#	29.18±1.05#

n=8; (mean±S.D., g);

* *P* < 0.05 as compared with normal control group (NC).

# P < 0.05 as compared with diabetic control group (DC). DLCPP = diabetic mice+ low dose CPP; DHCPP = diabetic mice + high dose CPP; DGLI = diabetic mice + glibencamide.

**Table 2.** Effect of CPP on blood glucose Level (mmol/L) in mice.

Groups	Number of animals	Days after dosing (day)				
		0	7	14	21	28
NC	8	5.15±0. 0.17	5.14±0.17	5.10±0.13	5.04±0.08	5.16±0.10
DC	8	15.36±0.39*	15.33±0.26*	15.33±0.35*	15.15±0.15*	15.23±0.29*
DLCPP	8	15.25±0.30*	12.67±0.24*#	11.29±0.28*#	10.56±0.32*#	9.76±0.29*#
DHCPP	8	15.39±0.28*	11.92±0.26*#	10.48±0.30*#	9.47±0.29*#	8.33±0.17*#
DGLI	8	15.29±0.29*	9.06±0.14*#	7.24±0.21*#	6.47±0.24*#	5.68±0.33*#

n=8; (mean±S.D., g);

* P < 0.05 as compared with normal control group.;

#. P < 0.05 as compared with diabetic control group.

**Table 3.** Effect of CPP on blood lipids (mmol/L) in mice.

Groups	Number of animals	TG	TC	HDL-c
NC	8	1.64±0.02	2.70±0.02	1.64±0.01
DC	8	2.05±0.04*	3.34±0.04*	0.77±0.02*
DLCPP	8	1.86±0.02*#	3.16±0.02*#	0.84±0.02*#
DHCPP	8	1.77±0.02*#	3.12±0.02*#	1.11±0.03*#
DGLI	8	1.72±0.01*#	3.03±0.05*#	1.39±0.02*#

n=8; (mean±S.D., g);

*. P<0.05 as compared with normal control group (NC);

#. P<0.05 as compared with diabetic control group.
